# Mathematical model of distal radius orientation

**DOI:** 10.3389/fsurg.2022.1000404

**Published:** 2022-10-14

**Authors:** Cheng-Kuang Chen, Tai-Yin Wu, Yu-Ciao Liao, Chiou-Shann Fuh, Kuan-Hao Chen, Pei-Wei Weng, Jr-Yi Wang, Chih-Yu Chen, Yu-Min Huang, Chung-Pei Chen, Yo-Lun Chu, Kuei-Lin Yeh, Ching-Hsiao Yu, Hung-Kang Wu, Wei-Peng Lin, Tsan-Hon Liou, Mai-Szu Wu, Chen-Kun Liaw

**Affiliations:** ^1^ Department of Orthopedics, Shin Kong Wu Ho-Su Memorial Hospital, Taipei, Taiwan; ^2^ Department of Biomedical Engineering, National Taiwan University, Taipei, Taiwan; ^3^ Department of Family Medicine, Zhongxing Branch, Taipei City Hospital, Taipei, Taiwan; ^4^ Institute of Epidemiology and Preventive Medicine, National Taiwan University, Taipei, Taiwan; ^5^ General Education Center, University of Taipei, Taipei, Taiwan; ^6^ Institute of Computer Science and Information Engineering, National Taiwan University, Taipei, Taiwan; ^7^ Department of Orthopedics, School of Medicine, College of Medicine, Taipei Medical University, Taipei City, Taiwan; ^8^ Department of Orthopedics, Shuang Ho Hospital, Taipei Medical University, New Taipei City, Taiwan; ^9^ Graduate Institute of Biomedical Optomechatronics, College of Biomedical Engineering; Research Center of Biomedical Device, Taipei Medical University, Taipei City, Taiwan; ^10^ International Ph.D. Program in Biomedical Engineering, College of Biomedical Engineering, Taipei Medical University, Taipei, Taiwan; ^11^ Department of Orthopedics, Cathay General Hospital, Taipei, Taiwan; ^12^ School of Medicine, College of Medicine, Fu Jen Catholic University, New Taipei City, Taiwan; ^13^ Department of Orthopaedics, Ditmanson Medical Foundation Chia-Yi Christian Hospital, Chia-Yi City, Taiwan; ^14^ Department of Long-Term Care and Management, WuFeng University, Chiayi County, Taiwan; ^15^ Department of Orthopaedic Surgery, Taoyuan General Hospital, Ministry of Health and Welfare, Taoyuan City, Taiwan; ^16^ Department of Orthopaedic Surgery, National Taiwan University Hospital, Taipei, Taiwan; ^17^ Department of Nursing, Yuanpei University of Medical Technology, Hsinchu City, Taiwan; ^18^ Department of Orthopedics, Postal Hospital, Taipei, Taiwan; ^19^ Department of Physical Medicine and Rehabilitation, School of Medicine, College of Medicine, Taipei Medical University, Taipei City, Taiwan; ^20^ Division of Nephrology, School of Medicine, College of Medicine, Taipei Medical University, Taipei City, Taiwan; ^21^ TMU Biodesign Center, Taipei Medical University, Taipei, Taiwan

**Keywords:** distal radius volar tilt, distal radius inclination, supination, pronation, rotation matrix

## Abstract

Distal radius orientation is important in evaluating Colles' fracture. In most cases, the wrist was protected by a bandage, splint, or cast. Therefore, it was difficult for the radiology technician to take perfect anteroposterior and lateral view radiographs. In this study, we build a mathematical model and calculate the pronation angle needed to produce dorsal tilt, which is a volar tilt in a perfect lateral view radiograph. The formulas are all incorporated into Excel to facilitate usage.

## Introduction

Reduction and the indications for the operation of Colles' fracture usually require the guidance of x-rays. These x-rays include wrist anteroposterior and lateral views evaluating volar (palmar) tilt and radial inclination (1–4). These two parameters are in fact three-dimensional. We see patients with dorsal tilt in some lateral view radiographs and volar tilt in others frequently.

**Figure 1:**
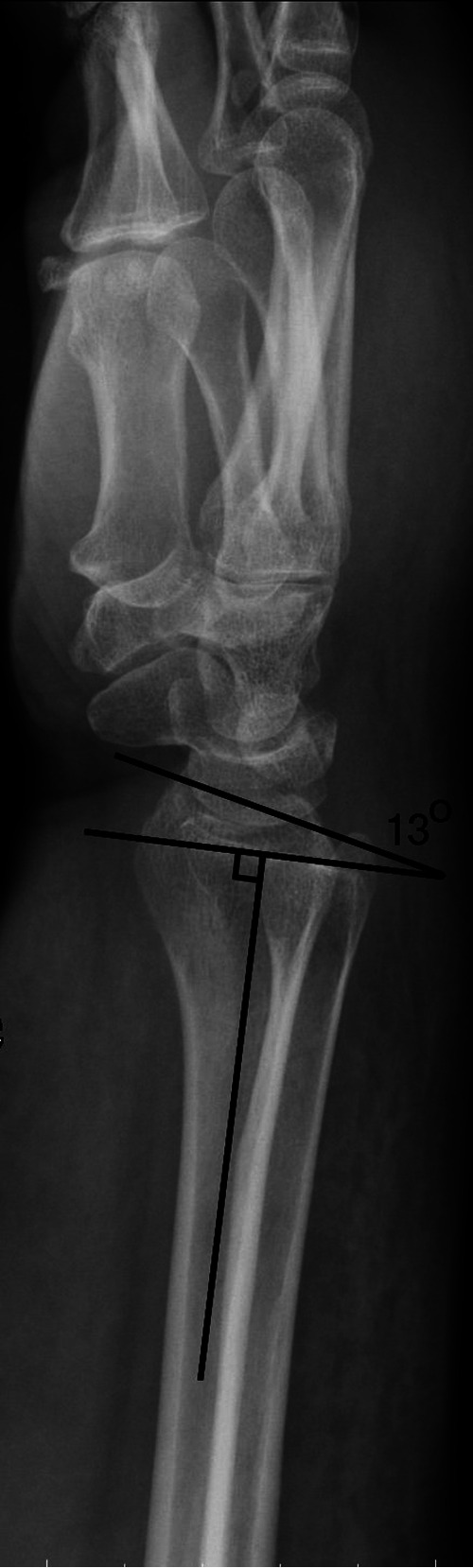
Wrist lateral view of a healthy Normal radius which showed dorsal tilt.

Figure 1 showed a patient with normal anatomy. However, the lateral view showed dorsal tilt.

These findings raised some questions:

1. What is the mathematical model of distal radius?
2. When does the lateral view show dorsal tilt in normal distal radius?

## Methods

The orientation of distal radius cartilage is three-dimensional. We must have a spatial concept when measuring it. Usually, it is presented by volar tilt and radial inclination. Using vector mathematics, we can present its orientation with a normal vector. First, we assume a 3D coordinate system.

**Figure 2:**
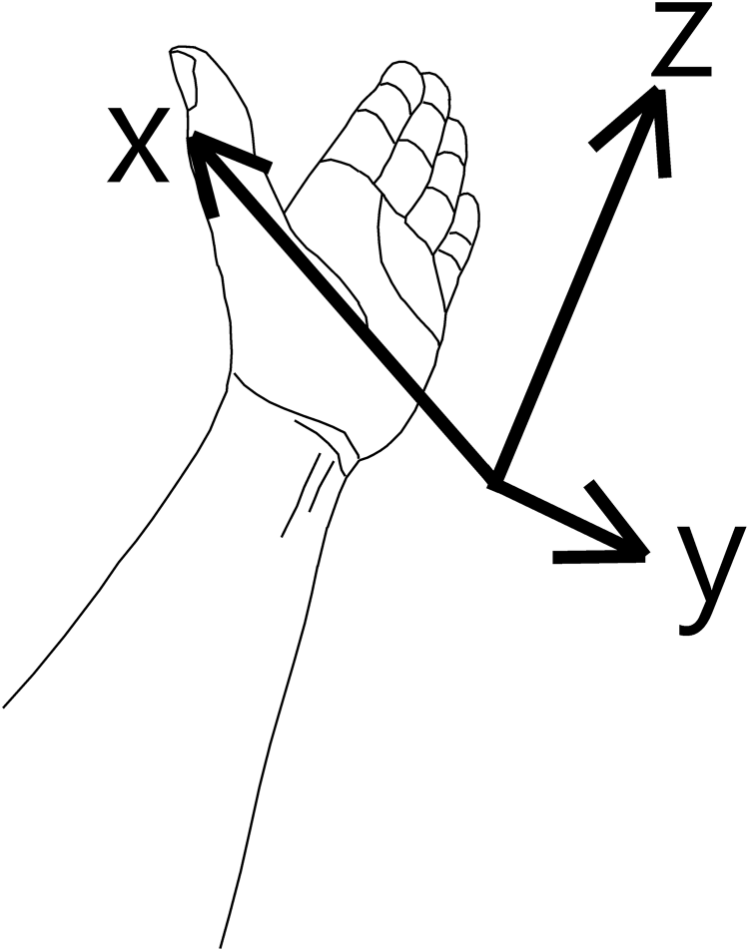
3D coordinate system we used in this study.

The *z*-axis is aligned by the axis of the radial shaft and directed from the elbow toward the distal radius. The *x*-axis is directed from the ulnar side toward the radial side. The *y*-axis is from the dorsal toward the volar side (Figure 2). Volar tilt is *θ*. The inclination is *ψ*.

Now we can get the distal radius orientation vector by trigonometric mathematics with the same principle (5).

Distal radius orientation normal vector (unit vector) = (−cosθsinφ,sinθcosφ,cosθcosφ)

In clinical practice, we usually take x-rays with some rotation around the *z*-axis, either supination or pronation. Thus, we use the rotation matrix to simulate this situation,

$$Rz(a)=\left[\begin{array}{ccc} \cos(a) & -\sin(a) & 0\\ \sin(a) & \cos(a) & 0\\ 0 & 0 & 1 \end{array}\right]$$

where “*a*” =  rotation around the *z*-axis or pronation angle. A positive value means pronation and negative means supination.

Thus, the normal vector after rotation “*a*” angle around the *z*-axis becomes the multiplication of the two matrixes,

$$\left[\begin{array}{ccc} \cos(a) & -\sin(a) & 0\\ \sin(a) & \cos(a) & 0\\ 0 & 0 & 1 \end{array}\right]\left[\begin{array}{c} -\cos\;\theta \;\sin\;\varphi \\ \sin\;\theta \;\cos\;\varphi \\ \cos\;\theta \;\cos\;\varphi \end{array}\right]$$

And the results after multiplication are

$$\begin{array}{r} \mathrm{Distal}\;\mathrm{radius}\;\mathrm{orientation}\;\mathrm{normal}\;\mathrm{vector}(\mathrm{unit}\;\mathrm{vector})=\\ (-\cos\;\theta \;\sin\;\varphi \;\cos\;a-\sin\;\theta \;\cos\;\varphi \;\sin\;a,\\ \;-\cos\;\theta \;\sin\;\varphi \;\sin\;a+\sin\;\theta \;\cos\;\varphi \;\cos\;a,\;\cos\;\theta \;\cos\;\varphi ) \end{array}$$

After pronating rotation “*a*” angle, the volar tilt becomes

Volar tilt after pronating rotation “*a*”
  =tan^−1^ (Y component on normal vector/Z component of the normal vector)
  =tan^−1^ ((−cos⁡θsinφsina+sinθcosφcosa)/cosθcosφ)).

**Figure 3:**
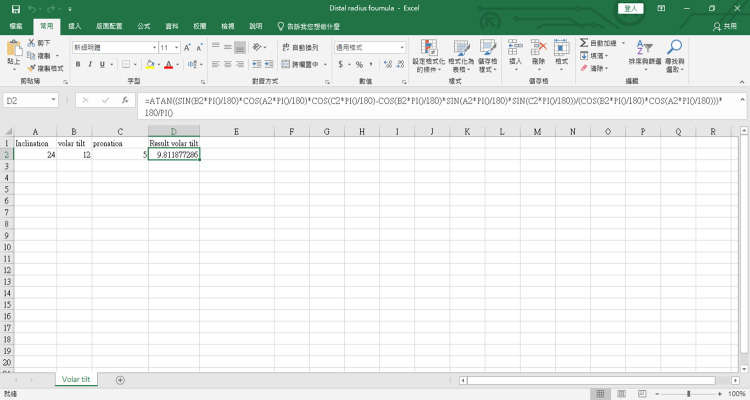
The excel program for the mathematical formula. The source code is as follows: “=ATAN((SIN(B2*PI()/180)*COS(A2*PI()/180)*COS(C2*PI()/180)-COS(B2*PI()/180)*SIN(A2*PI()/180)*SIN(C2*PI()/180))/(COS(B2*PI()/180)*COS(A2*PI()/180)))*180/PI()”. Readers can copy and paste the code directly.

This formula is incorporated into the Excel file (attachment file) (Figure 3.)

Because cos⁡θcos⁡φ>0,
Thus if (−cosθsinφsina+sinθcosφcosa)>0, it is volar tilt;
  if ((−cosθsinφsina+sinθcosφcosa)<0, it is dorsal tilt.

Then, we get the following:

If tanθ>tanφtana, it is volar tilt;
if tanθ<tanφtana, it is dorsal tilt.

or

if tana<tanθcotφ, it is volar tilt;
if tana>tanθcotφ, it is dorsal tilt.

We then get the result as follows:

If pronation (a) $>\mathrm{ta}n^{-1}\;\tan\;\theta \;\cot\;\varphi$), it changed from volar tilt to dorsal tilt. We incorporated it into excel. The excel file is attached.

We also incorporated the formula “pronation = tan^−1^ (tanθcotφ))” into the Excel file (supplementary material).

If we input the normal parameters, inclination 24°, and volar tilt 12°, we get the result. It showed 25.5°, which means if the patient pronates its wrist larger than 25.5°, the x-ray will show dorsal tilt.

## Results and discussion

We considered the distal radius articular surface a plane and built a mathematical model of its normal vector. We used this model and calculated the pronation angle, 25.5°, needed to have dorsal tilt in normal patients.

Its clinical importance cannot be overlooked. In practice, Orthopedics doctors can have every radiograph in perfect projection. We should always keep in mind that the radiographs are in fact three-dimensional.

In Figure 1, there are two solid pieces of evidence of pronation. First, the radial styloid is moved toward the volar side. Second, the ulna is moved toward the dorsal side.

In clinical practice, excessive pronation on the lateral radiograph of the wrist usually happened during the postoperative examination while excessive bandage (with splint or cast) confused the radiology technician. The confusing results also perplexed the surgeon. Our results can solve this clinical problem.

## Conclusions

We build a mathematical model for evaluating distal radius orientation and we found that the volar tilt will become dorsal tilt if the pronation is larger than 25.5°. Further study may be needed to determine precision.

## Data Availability

The original contributions presented in the study are included in the article/Supplementary Material, further inquiries can be directed to the corresponding author/s.
